# The updated genome of the Hungarian population of *Aedes koreicus*

**DOI:** 10.1038/s41598-024-58096-6

**Published:** 2024-03-30

**Authors:** Nikoletta Andrea Nagy, Gábor Endre Tóth, Kornélia Kurucz, Gábor Kemenesi, Levente Laczkó

**Affiliations:** 1 Department of Evolutionary Zoology and Human Biology, University of Debrecen, Debrecen, Hungary; 2 HUN-REN-UD Behavioural Ecology Research Group, University of Debrecen, Debrecen, Hungary; 3 Institute of Metagenomics, University of Debrecen, Debrecen, Hungary; 4 National Laboratory of Virology, Szentágothai Research Centre, University of Pécs, Pecs, Hungary; 5 Bernhard Nocht Institute for Tropical Medicine, WHO Collaborating Centre for Arbovirus and Hemorrhagic Fever Reference and Research, Hamburg, Germany; 6 Institute of Biology, Faculty of Sciences, University of Pécs, Pecs, Hungary; 7 HUN-REN-UD Conservation Biology Research Group, University of Debrecen, Debrecen, Hungary; 8 One Health Institute, University of Debrecen, Debrecen, Hungary

**Keywords:** *Aedes*, Invasive mosquito, Genome assembly, Third generation sequencing, Hybrid assembly, Functional annotation, Genome, Genome informatics

## Abstract

Vector-borne diseases pose a potential risk to human and animal welfare, and understanding their spread requires genomic resources. The mosquito *Aedes koreicus* is an emerging vector that has been introduced into Europe more than 15 years ago but only a low quality, fragmented genome was available. In this study, we carried out additional sequencing and assembled and characterized the genome of the species to provide a background for understanding its evolution and biology. The updated genome was 1.1 Gbp long and consisted of 6099 contigs with an N50 value of 329,610 bp and a BUSCO score of 84%. We identified 22,580 genes that could be functionally annotated and paid particular attention to the identification of potential insecticide resistance genes. The assessment of the orthology of the genes indicates a high turnover at the terminal branches of the species tree of mosquitoes with complete genomes, which could contribute to the adaptation and evolutionary success of the species. These results could form the basis for numerous downstream analyzes to develop targets for the control of mosquito populations.

## Introduction

Every year, vector-borne diseases are responsible for more than 700,000 deaths and account for more than 17% of all infectious diseases^1^. Vector-borne diseases (VBDs) pose a significant threat to global health, putting more than 80% of the world’s population at potential risk. Among these diseases, mosquito-borne diseases (MBDs) are the most important and significant contributor to this burden^2^. For many mosquito-borne diseases, an increase in incidence and geographical spread can be observed. A prime example is dengue fever, whose global incidence has increased 30-fold in the last five decades and which occurs in previously unaffected countries^3–5^. This phenomenon leads to the emergence of diseases in previously unaffected regions and their re-emergence in areas where they were previously eradicated. This process is largely driven by anthropogenic effects (globalization, deforestation, overpopulation, land use, etc.) and has a strong impact on MBDs^6–8^.

The introduction of mosquitoes from Asia has attracted attention in Europe^9^. In recent decades, numerous invasive mosquito species of the genus *Aedes* (*albopictus, japonicus*) have been introduced and have become successfully established. This has led to considerable distress to the population since they can act as potential vectors for exotic and native pathogens^10^. The first detection of *Aedes koreicus*, a potential vector of arboviruses^11^, outside its native range was in 2008 in an industrial area in Maasmechelen, Belgium, where this mosquito species is now firmly established and can overwinter^12^. Despite its continuous presence, *Ae. koreicus* has managed to colonize the surrounding areas to a limited extent^13^. In 2011, this species was found in north-eastern Italy, more precisely in the province of Belluno in the Veneto region, and rapidly expanded its range over the following ten years. It infested neighboring provinces and spread to more distant regions in northern Italy^14–17^. A revision of *Aedes japonicus* specimens collected in Slovenia confirmed the introduction of *Ae. koreicus* in 2013^18^. In 2015, the species appeared in southern Germany^19^. At the same time, *Ae. koreicus* was found in southwestern Hungary, where it established an overwintering but localized population^20,21^. More recently, *Ae. koreicus* has been detected in western Austria^22^, on the southern coast of the Crimean peninsula^23^ and in the Republic of Kazakhstan^24^. The literature indicates that, *Ae. koreicus* is a novel vector on the European continent^25^. In the field of mosquito invasion genomics, a fundamental and widely pursued goal is to elucidate the origins of invasive populations. Given their high propensity for invasion and associated disease risks, *Aedes* mosquitoes in particular have received much attention^26,27^.

The application of whole genome sequencing in mosquitoes has provided invaluable insights into fundamental biological processes at the molecular level and improved our understanding of their intricate mechanisms. Furthermore, this approach holds great promise for use in mosquito control strategies and the prevention of mosquito-borne disease transmission^28,29^. In the context of mosquito control, insecticide resistance is a major global challenge and an example of an extreme manifestation of adaptive evolution driven by human activities. Resistance in mosquitoes is often detected by bioassays or targeted sequencing methods^30^. An important application of population genomics throughout the development of the field has been understanding the evolution of insecticide resistance.

Invasive *Aedes* species can impact local ecosystems^31^. Genomic resources also help assess the impact of these invasive species. The increasing introduction of invasive species can pose a major challenge to local ecosystem functions^32^. Using genomic approaches, we can provide markers to monitor population genomes and invasion processes to assess whether invasiveness can be predicted from genome sequences^32,33^ to mitigate the impact of invasive species on ecosystems while reducing the likelihood of the spread of vector-borne diseases.

Given the spread of *Ae. koreicus* described above, accurate genomic characterization of the species could be important for numerous applications. In this study, we describe the improved genome sequence of *Aedes koreicus* (NCBI Assembly: GCA_024533555.1), focusing on the Hungarian population, that we assembled using the publicly available data (Sequence Read Archive: SRR14975285, SRR14975286) of Kurucz et al. (2022)^25^ supplemented with newly generated third-generation sequencing data. In addition to improving the assembly, we annotated the genome with a particular focus on genes that may be involved in insecticide resistance.

## Results and discussion

In this study, we used a hybrid genome assembly approach and combined Illumina short-read with Oxford Nanopore long-read sequencing data to reconstruct the high-quality draft genome of *Aedes koreicus*. Of the 87.31Gbp raw short-read sequencing data, 59.71 Gbp (489,687,726 reads) passed quality filtering with an average read length of 132 bp. A total of 8,196,976 reads with a base count of 37.90 Gbp were retained in the quality filtering of the long-read sequencing libraries. The read N50 values of the individual libraries ranged from 5923 to 7017 (mean = 6415 bp), with all libraries having an average read quality of 14.4. The three long-read libraries had a total throughput of 7.15 to 18.5 Gbp (mean = 12.6 Gbp) and yielded a total of 37.9 Gbp of sequencing data.

**Figure 1:**
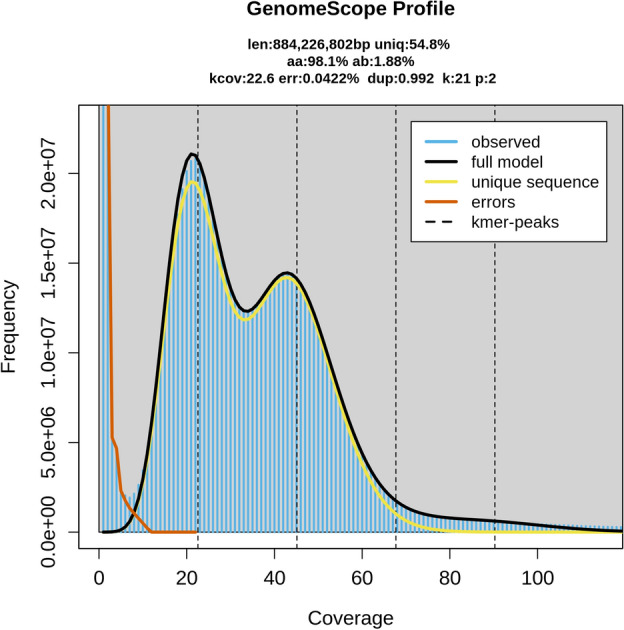
*K*-mer frequency histogram and genome characteristics as assessed with GenomeScope2.

Based on the 21-mer frequency of the short reads, GenomeScope2 estimated the genome size to be 884.23 Mbp with a unique *k*-mer frequency of 54.8% and a relatively high heteroziygosity rate (1.86%), regardless of the *k*-mer coverage threshold (Fig. 1). In contrast, CovEst estimated the genome size to be 1.49 Gbp. This 1.6-fold difference is most likely due to the different approaches of the two tools. GenomeScope2 accounts for variation in coverage by fitting negative binomials to the *k*-mer coverage histogram, but does not fit binomials for regions that occur more than twice. CovEst assumes uniform coverage and estimates genome size by dividing the total amount of sequencing data by the observed coverage. The contrast in estimated genome size is most likely due to pooling of individuals to obtain enough DNA for genome sequencing and/or sequencing of a highly repetitive genome (as confirmed by masking of repeats in the assembly). Given the assembly size (see below), which was filtered multiple times to remove duplicated contigs, the true genome size of the species should be between the two estimates and given the BUSCO score, closer to the result of CovEst.

**Figure 2:**
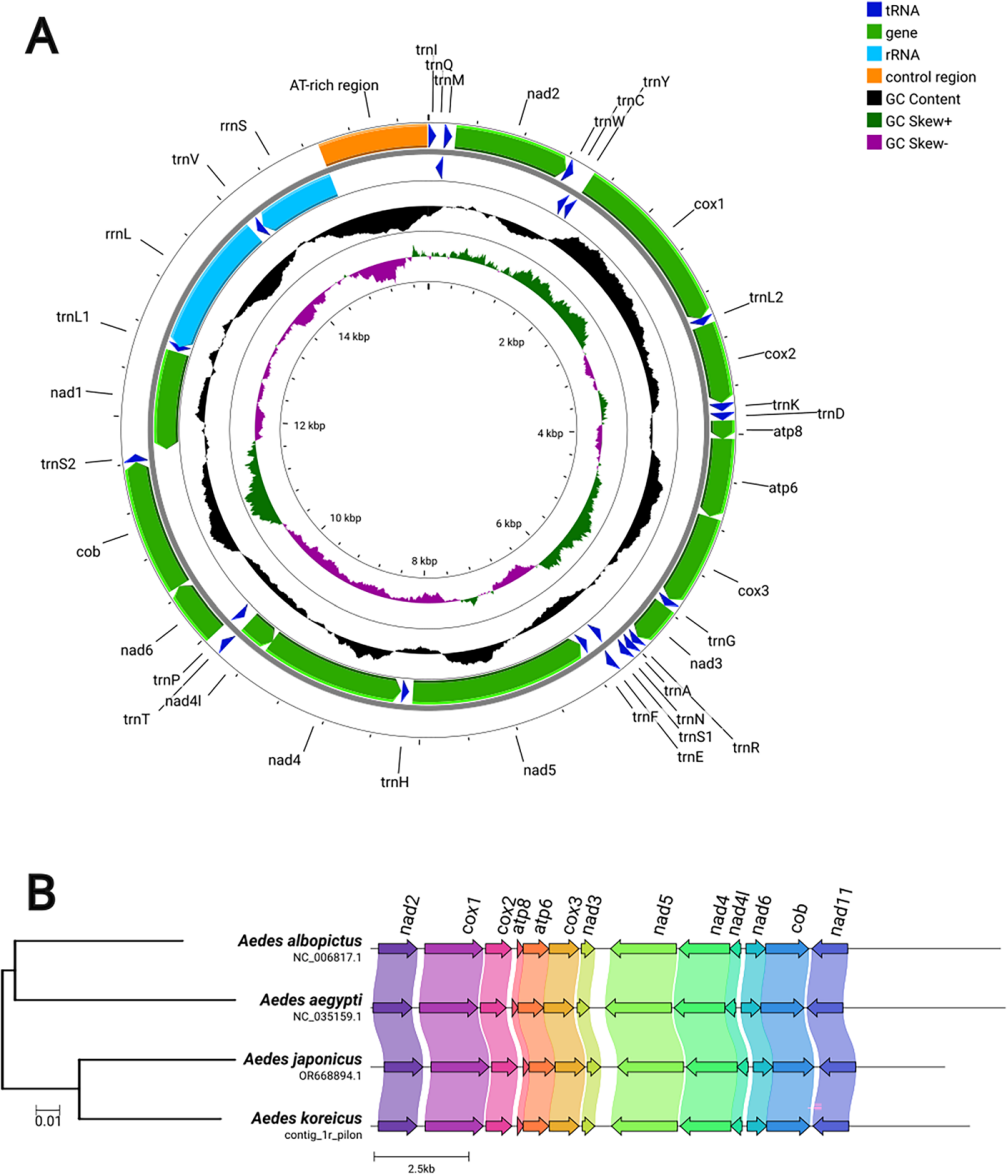
The circularized mitochondrial genome visualized by Proksee (**A**) and the structural comparison of the mitochondrial genomes of *Aedes* species with available complete mitochondrial genomes (**B**).

The mitochondrial genome appeared to be circular and 15,851 bps long, with a structure characteristic of the Culicidae family (Fig. 2A). The order and orientation of the genes of *Ae. koreicus*, *Aedes japonicus*, *Aedes aegypti* and *Aedes albopictus* matched perfectly. The result of skmer clustered *Ae. koreicus* together with *Ae. japonicus* as the sister clade of *Ae. aegypti* and *Ae. albopictus* (Fig. 2B). Before nuclear genome assembly, we excluded 0.26% of the short reads and 0.18% of the long reads for being mitochondrial; thus, 488,392,324 short reads and 8,182,409 long reads were used for nuclear genome assembly.

**Figure 3:**
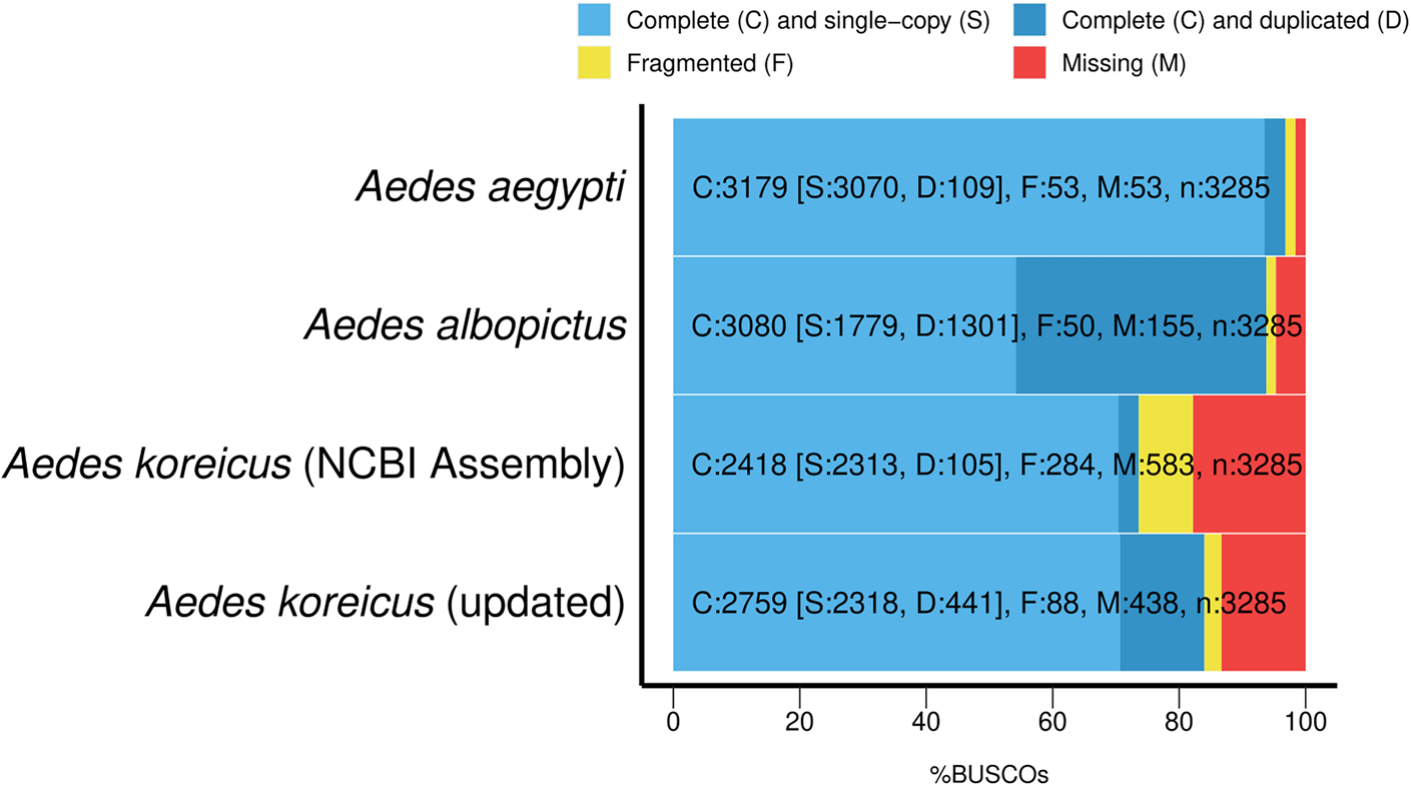
Comparison of completeness of *Aedes* genomes as output by BUSCO, including the previous version of the genome of *Ae. koreicus* (GCA_024533555.1).

The final assembly of the nuclear genome consisted of 6099 contigs with a total length of 1.10 Gbp, excluding all contigs flagged as contaminants (0.25% of the assembly). Compared to the previous version of the species’ genome (GCA_024533555.1), the number of contigs was reduced by one tenth, the N50 value increased from 18,623 bp to 329,610 bp and the L50 value decreased from 12,967 to 896 (Table 1 and Supplementary Table 1). The GC content of the new assembly (39.67%) remained unchanged compared to the previous version of the genome (39.7%). The ratio of complete BUSCOs increased by 10.4% and the ratio of missing BUSCOs decreased by 4.5% (Table 1). At the same time, we were able to increase the proportion of single-copy BUSCOs by 0.2%, suggesting several newly identified BUSCOs are duplicated in the genome (Fig. 3). Usually, such a high proportion of duplicates indicates duplicated contigs in the assembly, but given the multiple approaches we used to exclude false duplicates (i.e., pseudohaploid and redundans with multiple thresholds), these duplicates might actually exist in the species’ genome. However, the high proportion of duplicated BUSCO genes is not exceptional within the genus, e.g. 39.6% in the representative genome assembly of *Ae. albopictus* (Fig. 3), and gene duplications can be frequently observed in *Aedes* (e.g. Waterhouse et al. 2008^34^). The other recently published analysis of the complete genome^35^ reported an N50 value of 190,716 bp with an assembly size of 1.24 Gbp, consisting of 21,315 scaffolds and a BUSCO score of 91% (Duplicate = 8%, Missing = 2%). The assembly presented here shows better contiguity, but a somewhat lower BUSCO score. The relatively high duplication ratio again indicates that numerous valid gene duplicates can be found in the genome of the species. The differences between the two assemblies could be due to the different heterogeneity of the pooled DNA isolates, the different assembly approach including the filtering of duplicate contigs and the not yet well characterized variability of the species.

**Table 1:** Comparison of the contiguity and completeness of the publicly available and the newly assembled genome of *Aedes koreicus*.

Assembly	Updated genome	Previous version of the genome (GCA_024533555.1)
Number of contigs (≥ 0 bp)	6099	65,546
Number of contigs (≥ 1000 bp)	6099	65,542
Number of contigs (≥ 5000 bp)	6099	53,063
Number of contigs (≥ 10,000 bp)	6094	32,216
Number of contigs (≥ 25,000 bp)	5853	8037
Number of contigs (≥ 50,000 bp)	4525	1275
Total length (≥ 0 bp)	1,100,025,007	879,671,010
Total length (≥ 1000 bp)	1,100,025,007	879,667,056
Total length (≥ 5000 bp)	1,100,025,007	842,051,266
Total length (≥ 10,000 bp)	1,099,986,680	687,323,036
Total length (≥ 25,000 bp)	1,094,856,870	311,739,686
Total length (≥ 50,000 bp)	1,045,717,258	85,528,597
Largest contig	3,269,480	237,135
Total length	1,100,025,007	868,254,568
GC (%)	39.7	39.7
N50	329,610	18,859
N90	74,757	7248
auN	472,147	24,836
L50	896	13,697
L90	3626	43,206
# N’s per 100 kbp	3	0
Complete BUSCOs (%)	84	73.6
Single-copy BUSCOs (%)	70.6	70.4
Duplicated BUSCOs (%)	13.4	3.2
Fragmented BUSCOs (%)	2.7	8.6
Missing BUSCOs (%)	13.3	17.8

**Figure 4:**
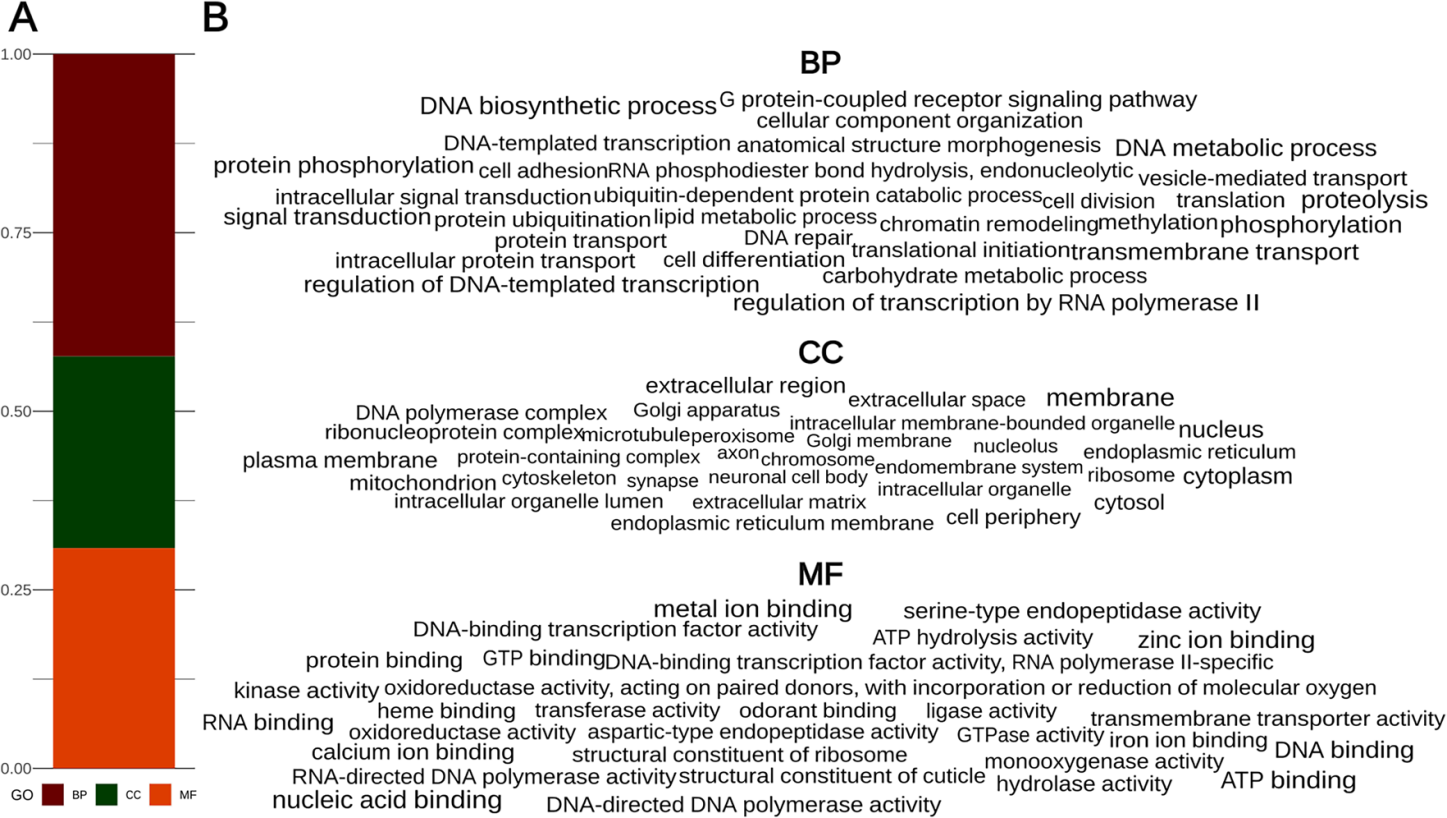
The ratio of functionally annotated protein coding genes assigned to the given GO term (**A**) and a wordcloud showing the 30 most abundant function by GO terms (**B**).

We soft-masked 60.62% of the genome as repetitive prior to gene prediction (1,256,253 identified repetitive regions with an average length of 530.85 bp), which is close to our initial estimate of repeat ratio based on *k*-mer frequencies (Fig. 1) and approximately 10% lower than reported for the species genome^35^. The ab initio gene prediction identified 28,154 potential protein-coding genes, whereas the homology-based method identified 43,226 genes. Merging these two sets of putative CDSes resulted in 47,796 unique amino acid sequences, of which 22,580 could be functionally annotated (47%). In addition, we identified 86 rRNA and 791 tRNA sequences in the final assembly. This gene count is comparable to the number of protein-coding genes in the publicly available genomes of Culicidae (mean number of genes in the proteomes used for phylogenomic reconstruction: 23,183). 42.3% of the functionally annotated genes were responsible for biological processes (BP), 26.8% contributed to cellular components (CC) and 30.8% were assigned to the GO term molecular function (MF) (Fig. 4A). In the BP category, DNA biosynthetic process, proteolysis, DNA metabolic process, phosphorylation and transmembrane transport were the most frequently occurring functions. Most genes with the GO term CC received the free text annotation membrane, nucleus, cytoplasm, plasma membrane and extracellular regions. The most frequently occurring molecular functions were nucleic acid binding, metal ion binding, ATP binding, zinc ion binding and RNA binding. We did not detect any strikingly overrepresented features in the frequency of the 50 most abundant gene functions (Fig. 4B). At the same time, we identified 218 genes involved in odorant binding, which not only highlights the importance of odorants in the feeding of *Ae. koreicus*, but also provides potential targets for the control of this vector species. These targets should be investigated with a larger sample size to validate their structure and function and potentially suggest specific repellent molecules (see: Yan et al. (2022)^36^ and Tiwari and Sowdhamini (2023)^37^). Out of 27 potential insecticide resistance genes^35,38^, we were able to identify the homolog of *aael*012918, *ace*1, *cyp*6bb2, *ABCA*3, cuticle protein, cuticle protein CP14.6, *cyp*9j26, *cyp*9j28, *cyp*9j32, ketohexokinase, modifier of *mdg*4, muscle calcium channel subunit alpha-1, *nav*, potassium voltage-gated channel protein Shaker, *rdl*, sodium leak channel non-selective protein and uncharacterized LOC5575776 (Supplementary Table 2). The cuticular protein was found in three copies in the genome of *Ae. aegypti* and in two copies in the genome of *Ae. koreicus* with free-text annotation “Cuticular protein 73” and “Pupal cuticle protein 78e”. Ketohexokinase could be annotated after ab initio gene prediction as “Phospholipid/glycerol acyltransferase domain-containing protein”. LOC5575776 appeared to be chitin synthase that was also described by Catapano et al. (2023)^35^ as a potential resistance gene. The cytochrome *cyp*9j26 was identified as a potential coding sequence by structural annotation, but no function could be identified using PANNZER (Supplementary Table 2). The remaining putative resistance genes, including *gstd*4, *gstd*6, multidrug resistance-associated protein 1 and multiple cytochromes, could not be identified, suggesting either low sequence similarity of the target sequences and/or that other mechanisms are involved in insecticide resistance in *Ae. koreicus*. In addition, in the functional annotation returned by PANNZER, we identified eight genes with the free text annotation “Deltamethrin resistance-associated NYD-OP7” and one with “Deltamethrin resistance protein prag01 domain-containing protein”, already described in *Culex pipiens*^39^. The genes labelled glutathione S-transferase (number of copies = 11) involved in insecticide resistance in several mosquito species^40^ all appeared to be putative, with the exception of delta-glutathione S-transferase (GST). Of the six copies of genes coding voltage-gated sodium channels, four appeared to be fragmented, and of the 11 copies of acetylcholinesterase, five were fragmented as indicated ‘(Fragment)’ in the gene annotation. Multidrug resistance-associated protein 1, which could not be identified by annotation transfer, could be found in three copies. We also discovered two copies of multidrug resistance-associated protein 7 and three copies of multidrug resistance-associated protein 9. Similarly to the odorant binding system, these potential target sequences should be investigated with a larger sample size and under experimental conditions to identify other genes involved in the insecticide resistance mechanisms of the species, e.g. with RNASeq.

**Figure 5:**
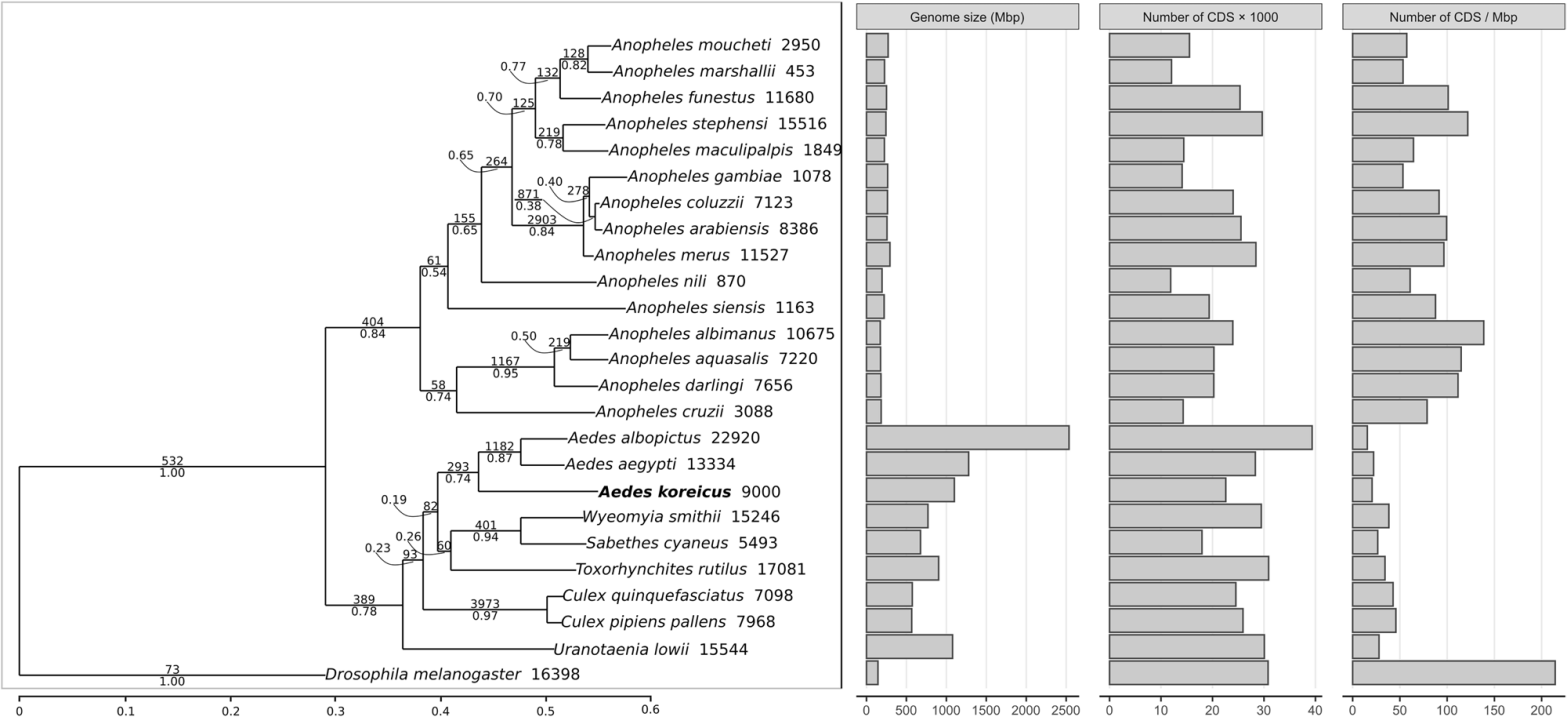
Phylogenomic reconstruction of Culicidae using *Drosophila melanogaster* as outgroup. The number of gene duplications and statistical robustness are given above each branch. Gene duplication events at terminal branches are given next to the species name. Panels next to the phylogenetic tree show the genome size, number of identified coding sequences (CDS) and CDS density of species.

OrthoFinder clustered all genes into 20,021 orthogroups, of which 4725 were contained in all species and 61 were single-copy orthologs. Of the 17,848 species-specific orthogroups, 403 (1134 genes) were specific to *Ae. koreicus*. The rooted species tree (Fig. 5) identified *D. melanogaster* as an outgroup and separated all *Anopheles* species from the genera *Aedes*, *Wyeomyia*, *Sabethes*, *Toxorhynchites*, *Culex* and *Uranotaenia*. Although the structure of the species tree was consistent with the phylogenetic reconstruction of Zadra et al. (2021)^41^ and Catapano et al. (2023)^35^, multiple branches received low support values, in particular the separation of *Culex* sp. and the separation of *Aedes* and its sister group consisting of *Wyeomyia*, *Sabethes* and *Toxorhynchites*. The structure within *Aedes* resembled the neighbor-joining phylogram reconstructed using pairwise mitochondrial (with *Ae. japonicus* missing from the phylogenetic reconstruction of nuclear genes due to the lack of genomic resources) distances, grouping *Ae. albopictus* and *Ae. aegypti* together and placing *Ae. koreicus* as sister to these species. The low support values indicate gene tree incongruence, which may result from ancient hybridization events that retain the correct topology of the phylogenetic tree but decrease phylogenetic support values^42^. As multiple phylogenetic reconstructions^35,41^ support a very similar phylogenetic hypothesis, a possible explanation for the low support could be that ancient hybridization played an important role in the speciation of the species group. At the same time, gene duplications were much more frequent in terminal branches (453–22,920, mean = 8852.64) than in internal branches (110–4281, mean = 1149) (Fig. 5), suggesting high gene turnover^43^, which can play a role in the adaptive strategy and evolutionary success of mosquito species. Genome size appeared to be much less variable in *Anopheles* than in the rest of the samples, and the genome size of *Ae. albopictus* was by far the largest, followed by *Ae. aegypti* and *Ae. koreicus*. The larger (*Aedes*) genomes contained a higher number of genes, resulting in a similar CDS density within the accessions of the genus (Fig. 5).

A single genome can hardly represent the entire variability of a species^44,45^. Multiple genome assemblies of the same species can be important to understand the unique aspects of the species’ biology^46^, including genome plasticity, identification of marker genes, and application of comparative genomic methods. In this particular case, the development of control strategies against this invasive species could benefit from multiple genomic resources that provide the opportunity to account for the variability of multiple genomes. Our results are consistent and comparable with another study conducted in parallel by Catapano et al. (2023)^35^. High-quality assemblies from different populations improve future genomic work on the global invasive populations of a species and facilitate the study of structural variation that may exist between different populations^47^. Furthermore, the question of whether invasiveness can be predicted by knowledge of genome variability^32^ can only be answered if we have multiple genomic resources at our disposal. *Aedes koreicus* is considered a vector on the rise^25^ with a continuous spread across Europe^13^. Therefore, future studies on this species should be conducted with international collaboration and shared resources to investigate the biology of this species and provide greater benefit to the scientific community.

In our study, we updated the first version of the *Aedes koreicus* genome assembly, aiming to create a well-characterized genome of the Hungarian populations that can be used as a resource for future studies on the diversity of genome structure and content of the species. Such genomic resources help to assess the impact and potential threat of invasive mosquito species and help designing specific control strategies^35^. The functional annotation presented here corroborates the presence of the majority of potential resistance genes in the genome reported previously^35^ and presents potential targets for the control of *Ae. koreicus* that could be evaluated experimentally.

## Methods

### Sample collection and genome sequencing

We collected mosquito larvae from stagnant waters, in the framework of a regular monitoring program run in urban and suburban areas of the city of Pécs (Hungary). Larvae were reared to adult stage in the laboratory and adult specimens were identified at the species level under a Nikon SMZ800N stereomicroscope (Minato, Japan) using morphological identification keys^48–50^. Four adult male *Aedes koreicus* specimens were captured on 23.06.2022 and pooled together. Nucleic acid was extracted from this pool using the DNeasy Blood & Tissue Kit (Qiagen, Germany) by following the manufacturer’s recommendations. We prepared two sequencing libraries using the Oxford Nanopore Sequencing Kit SQK-LSK110 (Oxford Nanopore, UK) and NEBNext FFPE Repair and Ultra II End Prep (New England Biolabs, USA) according to the protocol provided by the Nanopore Community. We used AMPure XP (Beckman Coulter, USA) magnetic beads for all purification steps and quantified the libraries using a Qubit Fluorometer v4 (Invitrogen, USA). Either the Small Fragment Buffer (SFB) or the Large Fragment Buffer (LFB) was used for size selection (the only difference between the libraries). Sequencing was performed with an Oxford Nanopore MinION MK1C (Oxford Nanopore, UK) sequencer using Flow Cells R9.4.1 (Oxford Nanopore, UK).

In addition to the sequencing libraries described above, we used the raw short (SRR14975286) and long (SRR14975285) sequencing datasets of the published genome^25^ to reconstruct the mitochondrial and nuclear genome of *Ae. koreicus*. The quality of Illumina short reads was checked using FastQC 0.11.9, and then adapters and low-quality bases were trimmed using fastp 0.20.1^51^. The parameters of fastp were set to trim sequences at both the 3’ and 5’ ends with a mean quality score of less than 15 using the default sliding window size and discard reads shorter than 90 bp (–cut_front 15 –cut_tail 15 –length_required 90). In addition, we turned on adapter detection for paired-end reads and enabled the polyX trimming at the 3’ ends (–detect_adapter_for_pe –trim_poly_x). To decrease the error rate, we corrected the sequencing errors using the *k*-mer frequency spectrum with Bloocoo 1.0.6^52^.

To achieve the highest possible read accuracy, we re-basecalled the raw reads used in Kurucz et al. (2022)^25^ with the same version of Guppy 6.5.7 (Oxford Nanopore Technologies, Oxford, UK) as for the newly generated data using the super-high accuracy basecall model. The quality of the long reads was checked using the R script MinIONQC 1.4.2^53^. We filtered and evaluated the quality of the long read sequences with NanoFilt 2.8.0^54^ and NanoPlot 1.40.0^54^.

We analyzed the 21-mer frequency spectrum in the filtered short-read dataset using KMC 3.1.1^55^ with the following parameters: minimum occurrence 1 (-ci1) and maximum frequency 10,000 (-cs10000). We used Genomescope 2.0^56^ to analyze the resulting k-mer histogram and estimate the genome size, *k*-mer coverage, heterozygosity and error rate of the sequencing data with different upper bounds of *k*-mer coverage (1000, 10,000, 100,000). In addition, we used CovEst 0.5.6^57^ assuming a repeat-rich genome (-m repeat) with the same *k*-mer histogram as input to confirm the estimated genome size.

### Mitochondrial genome assembly

Mitochondrial sequences are usually overrepresented in sequencing experiments^58,59^ and nuclear mitochondrial DNA segments (NUMT) are potentially present in the nuclear genome. Therefore, we first assembled the mitochondrial genome and used this assembly to exclude mitochondrial reads from the dataset to reduce the number of misassemblies in the nuclear genome and increase its contiguity^60^. We used the publicly available mitochondrial genome (GenBank accession number: NC_046946.1) as a reference for mapping short and long reads with BWA 0.7.17-r1188^61^ and Minimap2 2.17-r941^62^, respectively. In the case of short reads, reads with both ends were extracted with samtools 1.15.1^63^.

We performed mitochondrial de novo assembly using two software: GetOrganelle 1.7.6.1^64^ for short reads and Flye 2.9-b1768^65^ for long reads. The maximum number of extension rounds of GetOrganelle was set to 30 and the organelle type was set to animal mitochondrion (-R 30 -F animal_mt). In the case of Flye, we used an estimated genome size of 16,000 (assessed by the length of publicly available mitochondria of the genus *Aedes*) and set the coverage to 300 (-g 16 k –asm-coverage 300) to randomly resample the dataset and decrease the computational time required for the analysis. The two mitochondrial sequences were merged using quickmerge 0.3^66^. Sequence polishing consisted of three steps: we ran Racon 1.4.10^67^ and then medaka 1.7.2^68^ with the r941_min_sup_g507 model to create a more accurate consensus sequence of long reads; then we ran Pilon 1.23^69^ to correct SNPs and short indel variations using the alignment of the short read sequences.

We aligned both sequencing datasets to the polished mitochondrial genome and visualized the alignments using Integrative Genomics Viewer 2.16.0^70^ to ensure that there were no spurious segmental duplications in the assembly. Corrections to the consensus sequence were made manually in AliView 1.28^71^. We performed the functional annotation of the mitochondrion on the MITOS2 web server (http://mitos2.bioinf.uni-leipzig.de/index.py last accessed: June 9, 2023;^72^) and then visualized the genome with Proksee^73^. We used Clinker 0.0.27^74^ to assess whether there are structural variations in the mitochondrion using *Aedes japonicus* (OR668894.1)*, Aedes albopictus* (NC_006817.1) and *Aedes aegypti* (NC_035159.1) as reference taxa. The same four assemblies were used as input for skmer 3.3.0^75^ and the Jukes-Cantor-transformed genetic distances were visualized as a neighbor-joining phylogram using the pegas 1.2^76^ R 4.2.2^77^ package.

### Nuclear genome assembly

For the assembly of the nuclear genome, we first excluded reads that could be mapped to the mitochondrial genome. We excluded long reads with an alignment block length of at least 95% of their length and flagged all short reads with both ends mapped to the mitochondrial assembly as mitochondrial. Alignments were created using minimap2 in the same way as described above for the initial identification of mitochondrial reads. For the primary assembly, we used two different approaches: long reads were assembled using nextDenovo 2.5.0^78^, and the hybrid assembly with long and short reads was performed using MaSuRCA 4.0.5^79^. Both primary genome assemblies were polished following the same steps as for the mitochondrial sequence (see above). We checked the contiguity and completeness of the genomes with QUAST 5.0.2^80^ and BUSCO 5.2.2^81^ using the BUSCO gene set of the Diptera lineage from the Ortholog Database v10 (https://www.orthodb.org/).

Before merging the assemblies, we polished the assemblies again and then ran quickmerge 0.3 with the MaSuRCA assembly as hybrid and the nextDenovo assembly as self-assembly. Genome assembly of pooled samples may accumulate a high ratio of duplications; therefore, we removed false duplications in the polished sequences using create_pseudohaploid.sh (https://github.com/schatzlab/pseudohaploid/tree/master). Since we still found a relatively high ratio of duplications according to the results of BUSCO, we also ran redundans 0.13c^82^ with the assembly already curated with pseudohaploid as input. We ran redundans with different identity values (–identity 0.6, 0.65, 0.7, 0.75, 0.8, 0.85, 0.9, 0.95, 1.0) and overlap (–overlap 0.8, 0.85, 0.9, 0.95, 1.0) without scaffolding and gapclosing (–noscaffolding –nogapclosing) and chose the best parameters based on BUSCO results. We polished the reduced assembly again using the same approach as above, then identified possible contaminants with Bertax 0.1^83^ and excluded all sequences that were not classified as Arthropoda. The contiguity and gene completeness of the decontaminated assembly were checked again using QUAST and BUSCO.

### Gene prediction, functional annotation and phylogenetic reconstruction

We masked all repeat sequences, including tandem repeats and transposable elements in the genome with Red 2.0^84^ before gene prediction. We predicted transfer RNA (tRNA) and ribosomal RNA (rRNA) sequences using ARAGORN 1.2.38^85^ and Barrnap 0.9 (https://github.com/tseemann/barrnap), respectively. We predicted the sequence, location and structure of protein-coding genes in the soft-masked genome by combining ab initio and homology-based methods as implemented in the BRAKER 3.0.2 pipeline^86^. We carried out ab initio prediction with Augustus 3.5.0^87^. For homology-based gene prediction, we used arthropoda_odb11 to generate hints with ProtHint 2.6.0^88^, which were then used by GeneMark-EP 4.71_lic^88^ to generate the training gene set for Augustus. We performed homology-based prediction in two iterations and clustered coding sequences (CDS) to have the same protein product using CD-HIT 4.7^89^ with the following parameters: -c 1 -G 0 -aL 1.0 -aS 1.0 and then used the PANNZER2 [^90^ web server (http://ekhidna2.biocenter.helsinki.fi/sanspanz/; last accessed June 16, 2023) to functionally annotate the predicted genes, restricting the GO classes to arthropods.

To identify potential resistance genes, we transferred the annotations of the publicly available genome of *Aedes albopictus* (GCF_006496715.2) and checked if the target gene is present in the genome annotation of the de novo assembled genome of *Aedes koreicus*. We ran liftoff 1.6.3^91^ with the default settings, using the whole genome sequence and genome annotation of *Ae. albopictus* as the reference and the updated *Ae. koreicus* assembly as the target. Then, we searched for potential insecticide resistace genes (Supplementary Table 2) of *Ae. koreicus* in the transferred annotations. We used the targets reported by Djiappi-Tchamen et al. (2023)^38^, which were found in *Ae. albopictus* and *Ae. aegypti*, and the targets reported by Catapano et al. (2023)^35^, which are specific to *Ae. koreicus*. To verify the presence of genes, we used bedtools intersect 2.31.0^92^ to check whether the functional annotation of genomic regions with positive hits returned by PANNZER matched the function identified by annotation transfer.

We then searched for the orthologs of the functionally annotated genes of *Aedes koreicus* in other 23 species of the family Culicidae and used *Drosophila melanogaster* as an outgroup. We identified orthogroups and performed phylogenomic reconstruction of the species with OrthoFinder 2.5.5^93^ using the default settings. We used all accessions of Culicidae with available genome annotation in the NCBI genome database as of June 16, 2023 (Table 2).

**Table 2:** Species used for ortholog finding and phylogenetic analysis.

Species	Accession number	Protein count	Genome size (Mbp)
*Aedes aegypti*	GCF_002204515.2	28,317	1278.73
*Aedes albopictus*	GCF_006496715.2	39,354	2535.64
*Aedes koreicus*	this study	22,580	1100.03
*Anopheles albimanus*	GCF_013758885.1	23,947	172.60
*Anopheles aquasalis*	GCF_943734665.1	20,271	176.59
*Anopheles arabiensis*	GCF_016920715.1	25,532	256.82
*Anopheles coluzzii*	GCF_943734685.1	24,012	262.62
*Anopheles cruzii*	GCF_943734635.1	14,301	184.08
*Anopheles darlingi*	GCF_943734745.1	20,247	181.65
*Anopheles funestus*	GCF_943734845.2	25,342	250.71
*Anopheles gambiae*	GCF_000005575.2	14,102	265.03
*Anopheles maculipalpis*	GCF_943734695.1	14,422	224.07
*Anopheles marshallii*	GCF_943734725.1	12,038	225.73
*Anopheles merus*	GCF_017562075.2	28,438	294.38
*Anopheles moucheti*	GCF_943734755.1	15,528	271.32
*Anopheles nili*	GCF_943737925.1	11,869	195.24
*Anopheles sinensis*	GCA_000441895.2	19,352	214.50
*Anopheles stephensi*	GCF_013141755.1	29,660	243.46
*Culex pipiens pallens*	GCF_016801865.2	25,920	566.35
*Culex quinquefasciatus*	GCF_015732765.1	24,531	573.23
*Sabethes cyaneus*	GCF_943734655.1	17,957	676.04
*Toxorhynchites rutilus*	GCF_029784135.1	30,898	903.03
*Uranotaenia lowii*	GCF_029784155.1	30,072	1077.64
*Wyeomyia smithii*	GCF_029784165.1	29,479	769.23
*Drosophila melanogaster*	GCF_000001215.4	30,799	143.73

### Supplementary Information

Supplementary Information 1.

Supplementary Information 2.

## Data Availability

We deposited all newly generated data described in this study in the NCBI database under BioProject PRJNA728830. The raw data belonging to BioSample SAMN19104546 can be found in the Sequence Read Archive (SRA) database under accessions SRR27118085 and SRR27118086, whereas the updated genome assembly can be found in the Assembly database under accession GCA_024533555.2. This Whole Genome Shotgun project has been deposited at DDBJ/ENA/GenBank under the accession JAHHFK000000000. The version described in this paper is version JAHHFK020000000. The analysis schema is available as a supplementary file to this paper (Supplementary File 1). The structural and functional annotations of the assembly as well as the genome version presented in this study are made public in the Zenodo data repository under 10.5281/zenodo.10278300.
