# Supplementary material for: The updated genome of the Hungarian population of Aedes koreicus
Source: Sci Rep. 2024 Mar 30;14:7545. doi: 10.1038/s41598-024-58096-6 (PMC10981705; doi:10.1038/s41598-024-58096-6)
Supplement: Supplementary file 1 — Supplementary Information 1. [file 41598_2024_58096_MOESM1_ESM.pdf]

Workflow of the assembly and annotation of the genome of *Aedes koreicus*

Quality check and filter of the reads

Short reads

```
# Basic quality check (FastQC 0.11.9)

mkdir fastqc
fastqc -t 2 -o fastqc/ Aedes_R1.fq.gz Aedes_R2.fq.gz 2>
fastqc/fastqc.log

# Quality check and simultaneous filtering (fastp 0.20.1)

fastp -i Aedes_R1.fq.gz -I Aedes_R2.fq.gz -o Aedes_fastp_R1.fq.gz
-O Aedes_fastp_R2.fq.gz -w 12 -5 15 -3 15 --detect_adapter_for_pe
-x -f 15 -F 15 -t 2 -T 2 -l 90 -q 15 -j Aedes_fastp.json -h
Aedes_fastp.html 2> fastp.log

# Correcting sequencing error using the k-mer frequency spectrum
(Bloocoo 1.0.6)

Bloocoo -nb-cores 12 -file
Aedes_fastp_R1.fq.gz,Aedes_fastp_R2.fq.gz 1> bloocoo.stdout 2>
bloocoo.err

# Estimation of genome size, k-mer coverage, heterozigosity and
error-rate (KMC 3.1.1; Genomescope 2.0)

kmc -k21 -t12 -m64 -ci1 -cs10000 Aedes_fastp_cor_R1.fq.gz
Aedes_fastp_cor_R2.fq.gz reads tmp/ 1> kmc.log 2> kmc.err
kmc_tools transform reads histogram reads.histo -cx10000 & 1>
kmc_histo.log 2> kmc_histo.err

genomescope2 -i reads.histo -o gs2_out -p 2 -k 21 1> gs2.log 2>
gs2.err
```

Long reads

```
# Filtering and assessing the quality of the long read sequences
(NanoFilt 2.8.0; NanoPlot 1.40.0)

NanoFilt -q 7 -l 500 --headcrop 50 --tailcrop 50 | gzip &>
aedes_nanopore_filt.fq.gz
NanoPlot -t 12 --N50 --fastq aedes_nanopore_filt.fq.gz -o
aedes_nanopore_filt_nanoplot
```

Mitochondrial genome assembly

Subset mitochondrial reads

```
# Subset reads that align to the reference sequence (GenBank acc.
number: NC_046946.1)

# Subset short read pairs with both ends aligned (BWA 0.7.17-
r1188; samtools 1.15.1)

bwa mem -t 12 a_koreicus_mt.fa Aedes_fastp_cor_{R1,R2}.fq.gz 2>
bwa_mt.err | samtools view -h -b -u -@ 12 -F 12 > aedes_mt.bam
&& \
samtools fastq -1 aedes_mt_R1.fq -2 aedes_mt_R2.fq -s
aedes_mt_s.fq -0 aedes_mt_0.fq aedes_mt.bam 1> samtools_fastq.log
2> samtools_fastq.err && \
ls *fq | xargs -n 1 -P 4 gzip

# Subset long reads of which 95% can be aligned to the reference
mitochodrion (Minimap2 2.17-r941)

minimap2 -x map-ont -t 12 a_koreicus_mt.fa
aedes_nanopore_filt.fq.gz > aedes_mt.paf 2> minimap.err &&
cut -f 1,2,3,4,11 aedes_mt.paf | awk '{OFS="\t"} {$6 = $4 - $3}
{$7 = $6 / $2*100} {print $0}' | awk '$7 > 95' | cut -f 1 >
aedes_mt.list && \
zgrep -A 3 --no-group-separator -wf aedes_mt.list
aedes_nanopore_filt.fq.gz | gzip > aedes_nanopore_mt.fq.gz
```

Genome assembly

```
# Short read assembly (GetOrganelle 1.7.6.1)

get_organelle_from_reads.py -1 aedes_mt_R1.fq.gz -2
aedes_mt_R2.fq.gz -R 30 -F animal_mt -o aedes_illumina_mt -t 12
ls aedes_illumina_mt/*fq | xargs -n 1 -P 4 gzip

# Long read assembly (Flye 2.9-b1768)

flye --nano-raw aedes_nanopore_mt.fq.gz -o aedes_nanopore_flye_mt
-t 12 -g 16k --asm-coverage 300

# Merge the two assemblies (quickmerge 0.3)

merge_wrapper.py
aedes_illumina_mt/animal_mt.K115.scaffolds.graph1.1.path_sequence.
fasta aedes_nanopore_flye_mt/assembly.fasta
mv merged_out.fasta mt_merged.fa

# Polish the merged assembly (Minimap2 2.17-r941; Racon 1.4.10;
medaka 1.7.2; Pilon 1.23)

minimap2 -x map-ont -t 12 -a mt_merged.fa aedes_nanopore_mt.fq.gz
> aligned1.sam && \
```

```
racon -t 12 -f aedes_nanopore_mt.fq.gz aligned1.sam mt_merged.fa >
racon_assembly.fa

apptainer exec medaka.sif mini_align -i aedes_nanopore_mt.fq.gz -r
racon_assembly.fa -P -m -p racon_assembly -t 12 && \
apptainer exec medaka.sif medaka consensus --model
r941_min_sup_g507 --threads 2 racon_assembly.bam
racon_assembly.hdf && \
apptainer exec medaka.sif medaka stitch racon_assembly.hdf
racon_assembly.fa medaka_racon.fa --threads 2

bwa index medaka_racon.fa && \
bwa mem -t 12 medaka_racon.fa aedes_mt_{R1,R2}.fq.gz | samtools
view -h -b -u -@ 12 | samtools sort -@ 12 > medaka_racon.bam && \
sambamba markdup -t 12 -p medaka_racon.bam
medaka_racon_markdup.bam 1> markdup.log 2> markdup.err && \
apptainer exec pilon.sif pilon --genome medaka_racon.fa --frags
medaka_racon_markdup.bam --threads 12 --output pilon --fix
snps,indels,gaps,local &> pilon.log
mv pilon.fasta mt_merged_polished.fa

seqkit seq --seq-type DNA -p -r mt_merged_polished.fa >
mt_merged_polished_c.fa

# Manual correction to construct circular genome ->
mt_merged_polished_c_ed.fa
```

Nuclear genome assembly

Remove mitochondrial reads

```
# Exclude reads that align to the assembled mitochondrial genome
(BWA 0.7.17-r1188; samtools 1.15.1; Minimap2 2.17-r941)

bwa index mt_merged_polished_c_ed.fa
bwa mem -t 12 mt_merged_polished_c_ed.fa
Aedes_fastp_cor_{R1,R2}.fq.gz 2> bwa_mt_asm.err | samtools view -h
-b -u -F 12 -@ 96 | samtools sort -n > aedes_mt_mapped.bam && \
samtools view aedes_mt_mapped.bam | cut -f 1 > aedes_mt_sr.list

minimap2 -x map-ont -t 12 mt_merged_polished_c_ed.fa
aedes_nanopore_filt.fq.gz > aedes_mt.paf 2> minimap_mt_asm.err
&& \
cat aedes_mt.paf | cut -f 1,2,3,4,11 | awk '{OFS="\t"} {$6 = $4 -
$3} {$7 = $6 / $2*100} {print $0}' | awk '$7 > 95' | cut -f 1 >
aedes_mt_lr.list

zcat Aedes_fastp_cor_R1.fq.gz | awk 'NR%4 == 1' | cut -f 1 -d " "
| sed "s/^@//" > sr_read.names
zcat aedes_nanopore_filt.fq.gz | awk 'NR%4 == 1' | cut -f 1 -d " "
| sed "s/^@//" > lr_read.names
grep -vwf aedes_mt_sr.list sr_read.names > aedes_sr_nuc.list && \
grep -vwf aedes_mt_lr.list lr_read.names > aedes_lr_nuc.list && \
zgrep -A 3 -wf aedes_sr_nuc.list Aedes_fastp_cor_R1.fq.gz --no-
group-separator | gzip > aedes_nuc_R1.fq.gz &
zgrep -A 3 -wf aedes_sr_nuc.list Aedes_fastp_cor_R2.fq.gz --no-
group-separator | gzip > aedes_nuc_R2.fq.gz &
zgrep -A 3 -wf aedes_lr_nuc.list aedes_nanopore_filt.fq.gz --no-
group-separator | gzip > aedes_nanopore_nuc.fq.gz
```

Long read assembly

```
# (nextDenovo 2.5.0)
realpath aedes_nanopore_nuc.fq.gz > input.fofn
cat aedes_nd.cfg

[General]
job_type = slurm
job_prefix = aedes_nd
task = all
rewrite = yes
deltmp = yes
parallel_jobs = 20
input_type = raw
read_type = ont # clr, ont, hifi
input_fofn = input.fofn
workdir = aedes_nd

[correct_option]
read_cutoff = 0k
```

```
genome_size = 900m # estimated genome size
sort_options = -m 20g -t 15
minimap2_options_raw = -t 32
pa_correction = 3
correction_options = -p 32

[assemble_option]
minimap2_options_cns = -t 32
nextgraph_options = -a 1

nextDenovo aedes_nd.cfg
```

Hybrid assembly

```
# (MaSuRCA 4.0.5)
masurca -t 24 -i aedes_nuc_R1.fq.gz,aedes_nuc_R2.fq.gz -r
aedes_nanopore_nuc.fq.gz -o aedes_masurca
```

Polish assemblies

```
#(Minimap2 2.17-r941; Racon 1.4.10; medaka 1.7.2; Pilon 1.23)

# nextDenovo

minimap2 -x map-ont -t 24 -a aedes_nd/03.ctg_graph/nd.asm.fasta
aedes_nanopore_nuc.fq.gz > aligned1.sam
racon -t 24 -f aedes_nanopore_nuc.fq.gz aligned1.sam
aedes_nd/03.ctg_graph/nd.asm.fasta > racon_assembly.fa

apptainer exec medaka.sif mini_align -i aedes_nanopore_nuc.fq.gz -
r racon_assembly.fa -P -m -p racon_assembly -t 24
apptainer exec medaka.sif medaka consensus --model
r941_min_sup_g507 --threads 2 racon_assembly.bam
racon_assembly.hdf
apptainer exec medaka.sif medaka stitch racon_assembly.hdf
racon_assembly.fa medaka_racon.fa --threads 2

bwa index medaka_racon.fa
bwa mem -t 24 medaka_racon.fa aedes_nuc_{R1,R2}.fq.gz | samtools
view -h -b -u -@ 24 | samtools sort -@ 24 > medaka_racon.bam
sambamba markdup -t 24 -p medaka_racon.bam
medaka_racon_markdup.bam 1> markdup.log 2> markdup.err
apptainer exec pilon.sif java -jar -Xmx120g /pilon/pilon-1.23.jar
--genome medaka_racon.fa --frags medaka_racon_markdup.bam --
threads 24 --output pilon --fix snps,indels,gaps,local &>
pilon.log
mv pilon.fasta aedes_nuc_nd_polished.fa

# MaSuRCA

minimap2 -x map-ont -t 24 -a
masurca/CA.mr.79.17.15.0.02/primary.genome.scf.fasta
aedes_nanopore_nuc.fq.gz > aligned1.sam
```

```
racon -t 24 -f aedes_nanopore_nuc.fq.gz aligned1.sam
masurca/CA.mr.79.17.15.0.02/primary.genome.scf.fasta >
racon_assembly.fa

apptainer exec medaka.sif mini_align -i aedes_nanopore_nuc.fq.gz -
r racon_assembly.fa -P -m -p racon_assembly -t 24
apptainer exec medaka.sif medaka consensus --model
r941_min_sup_g507 --threads 2 racon_assembly.bam
racon_assembly.hdf
apptainer exec medaka.sif medaka stitch racon_assembly.hdf
racon_assembly.fa medaka_racon.fa --threads 2

bwa index medaka_racon.fa
bwa mem -t 24 medaka_racon.fa aedes_nuc_{R1,R2}.fq.gz | samtools
view -h -b -u -@ 24 | samtools sort -@ 24 > medaka_racon.bam
sambamba markdup -t 24 -p medaka_racon.bam
medaka_racon_markdup.bam 1> markdup.log 2> markdup.err
apptainer exec pilon.sif java -jar -Xmx120g /pilon/pilon-1.23.jar
--genome medaka_racon.fa --frags medaka_racon_markdup.bam --
threads 24 --output pilon --fix snps,indels,gaps,local &>
pilon.log
mv pilon.fasta aedes_nuc_masurca_polished.fa
```

Remove false duplications

```
# Remove false duplications originating from assembly errors
(pseudohaploid)
```

```
create_pseudohaploid.sh aedes_nuc_nd_polished.fa
aedes_nuc_masurca_pseudohap
create_pseudohaploid.sh aedes_nuc_masurca_polished.fa
aedes_nuc_masurca_pseudohap
```

Polish assemblies

```
# nextDenovo
```

```
minimap2 -x map-ont -t 24 -a aedes_nuc_nd_pseudohap.pseudohap.fa
aedes_nanopore_nuc.fq.gz > aligned1.sam
racon -t 24 -f aedes_nanopore_nuc.fq.gz aligned1.sam
aedes_nuc_nd_pseudohap.pseudohap.fa > racon_assembly.fa

apptainer exec medaka.sif mini_align -i aedes_nanopore_nuc.fq.gz -
r racon_assembly.fa -P -m -p racon_assembly -t 24
apptainer exec medaka.sif medaka consensus --model
r941_min_sup_g507 --threads 2 racon_assembly.bam
racon_assembly.hdf
apptainer exec medaka.sif medaka stitch racon_assembly.hdf
racon_assembly.fa medaka_racon.fa --threads 2

bwa index medaka_racon.fa
bwa mem -t 24 medaka_racon.fa aedes_nuc_{R1,R2}.fq.gz | samtools
view -h -b -u -@ 24 | samtools sort -@ 24 > medaka_racon.bam
```

```
sambamba markdup -t 24 -p medaka_racon.bam
medaka_racon_markdup.bam 1> markdup.log 2> markdup.err
apptainer exec pilon.sif java -jar -Xmx120g /pilon/pilon-1.23.jar
--genome medaka_racon.fa --frags medaka_racon_markdup.bam --
threads 24 --output pilon --fix snps,indels,gaps,local &>
pilon.log
mv pilon.fasta aedes_nuc_nd_pseudohap_polished.fa
```

```
# MaSuRCA
```

```
minimap2 -x map-ont -t 24 -a
aedes_nuc_masurca_pseudohap.pseudohap.fa aedes_nanopore_nuc.fq.gz
> aligned1.sam
racon -t 24 -f aedes_nanopore_nuc.fq.gz aligned1.sam
aedes_nuc_masurca_pseudohap.pseudohap.fa > racon_assembly.fa

apptainer exec medaka.sif mini_align -i aedes_nanopore_nuc.fq.gz -
r racon_assembly.fa -P -m -p racon_assembly -t 24
apptainer exec medaka.sif medaka consensus --model
r941_min_sup_g507 --threads 2 racon_assembly.bam
racon_assembly.hdf
apptainer exec medaka.sif medaka stitch racon_assembly.hdf
racon_assembly.fa medaka_racon.fa --threads 2

bwa index medaka_racon.fa
bwa mem -t 24 medaka_racon.fa aedes_nuc_{R1,R2}.fq.gz | samtools
view -h -b -u -@ 24 | samtools sort -@ 24 > medaka_racon.bam
sambamba markdup -t 24 -p medaka_racon.bam
medaka_racon_markdup.bam 1> markdup.log 2> markdup.err
apptainer exec pilon.sif java -jar -Xmx120g /pilon/pilon-1.23.jar
--genome medaka_racon.fa --frags medaka_racon_markdup.bam --
threads 24  --output pilon --fix snps,indels,gaps,local &>
pilon.log
mv pilon.fasta aedes_nuc_masurca_pseudohap_polished.fa
```

Merge the assemblies

```
# (quickmerge 0.3)
```

```
merge_wrapper.py aedes_nuc_nd_pseudohap_polished.fa
aedes_nuc_masurca_pseudohap_polished.fa
mv merged_out.fasta aedes_nuc_nd_masurca.fa
```

Polish merged genome

```
minimap2 -x map-ont -t 24 -a aedes_nuc_nd_masurca.fa
aedes_nanopore_nuc.fq.gz > aligned1.sam
racon -t 24 -f aedes_nanopore_nuc.fq.gz aligned1.sam
aedes_nuc_nd_masurca.fa > racon_assembly.fa

apptainer exec medaka.sif mini_align -i aedes_nanopore_nuc.fq.gz -
r racon_assembly.fa -P -m -p racon_assembly -t 24
```

```
apptainer exec medaka.sif medaka consensus --model
r941_min_sup_g507 --threads 2 racon_assembly.bam
racon_assembly.hdf
apptainer exec medaka.sif medaka stitch racon_assembly.hdf
racon_assembly.fa medaka_racon.fa --threads 2

bwa index medaka_racon.fa
bwa mem -t 24 medaka_racon.fa aedes_nuc_{R1,R2}.fq.gz | samtools
view -h -b -u -@ 24 | samtools sort -@ 24 > medaka_racon.bam
sambamba markdup -t 24 -p medaka_racon.bam
medaka_racon_markdup.bam 1> markdup.log 2> markdup.err
apptainer exec pilon.sif java -jar -Xmx120g /pilon/pilon-1.23.jar
--genome medaka_racon.fa --frags medaka_racon_markdup.bam --
threads 24 --output pilon --fix snps,indels,gaps,local &>
pilon.log
mv pilon.fasta aedes_nuc_nd_masurca_polished.fa
```

Remove false duplications

```
# (pseudohaploid)
create_pseudohaploid.sh aedes_nuc_nd_masurca_polished.fa
aedes_nuc_merged_pseudohap_polished.fa
```

Reduce the number of false duplications

```
# (redundans 0.13c)

ids="0.60 0.65 0.70 0.75 0.80 0.85 0.90 0.95 1.0"
ovls="0.80 0.85 0.90 0.95 1.0"

for i in $ids
do
for j in $ovls
do
redundans.py -f aedes_nuc_merged_pseudohap_polished.fa -t 36 -o
aedes_redundans_id${i}_ovl${j} --identity ${i} --overlap ${j} --
noscaffolding --nogapclosing
done
done
```

Check gene completeness

```
# (BUSCO 5.2.2)
for i in $ids
do
for j in $ovls
do
busco -m genome -c 36 -o aedes_redundans_id${i}_ovl${j}_busco -i
aedes_redundans_id${i}_ovl${j}/scaffolds.reduced.fa -l diptera
done
done
```

Final polish

```
# Final polish on the selected redundans output
```

```
minimap2 -x map-ont -t 24 -a
aedes_redundans_id1.0_ovl0.80/scaffolds.reduced.fa
aedes_nanopore_nuc.fq.gz > aligned1.sam
racon -t 24 -f aedes_nanopore_nuc.fq.gz aligned1.sam
aedes_redundans_id1.0_ovl0.80/scaffolds.reduced.fa >
racon_assembly.fa

apptainer exec medaka.sif mini_align -i aedes_nanopore_nuc.fq.gz -
r racon_assembly.fa -P -m -p racon_assembly -t 24
apptainer exec medaka.sif medaka consensus --model
r941_min_sup_g507 --threads 2 racon_assembly.bam
racon_assembly.hdf
apptainer exec medaka.sif medaka stitch racon_assembly.hdf
racon_assembly.fa medaka_racon.fa --threads 2

bwa index medaka_racon.fa
bwa mem -t 24 medaka_racon.fa aedes_nuc_{R1,R2}.fq.gz | samtools
view -h -b -u -@ 24 | samtools sort -@ 24 > medaka_racon.bam
sambamba markdup -t 24 -p medaka_racon.bam
medaka_racon_markdup.bam 1> markdup.log 2> markdup.err
apptainer exec pilon.sif java -jar -Xmx120g /pilon/pilon-1.23.jar
--genome medaka_racon.fa --frags medaka_racon_markdup.bam --
threads 24 --output pilon --fix snps,indels,gaps,local &>
pilon.log
mv pilon.fasta aedes_final_asm_polished.fa
```

Check genome characteristics and gene completeness

```
# (QUAST 5.0.2; BUSCO 5.2.2)
```

```
quast-lg.py -o aedes_final_asm_polished_quast -t 12
aedes_redundans_id1.0_ovl0.80/scaffolds.reduced.fa
aedes_final_asm_polished.fa

busco -m genome -c 36 -o aedes_final_asm_polished_busco -i
aedes_final_asm_polished.fa -l diptera
```

Contamination check

```
# (Bertax 0.1)
```

```
bertax --conf_matrix_file aedes_final_asm_bertax_conf.json
aedes_final_asm.fa > aedes_final_asm_bertax.tsv
```

```
# Remove non-arthropod sequences (SeqKit 2.3.0) and check the
characteristics and gene completeness
```

```
grep -v Arthropoda aedes_final_asm_bertax.tsv | cut -f 1 -d " " >
non_arthropoda.ids
seqkit grep -v -f non_arthropoda.ids aedes_final_asm.fa >
aedes_final_decontam_asm.fa
```

```
quast-lg.py -o aedes_final_decontam_asm_quast -t 12
aedes_redundans_id1.0_ovl0.80/scaffolds.reduced.fa
aedes_final_decontam_asm.fa

busco -m genome -c 36 -o aedes_final_decontam_asm_busco -i
aedes_final_decontam_asm.fa -l diptera
```

Genome annotation

Repeat masking

```
# (Red 2.0)

mkdir -p {red_in,red_out}
cp aedes_final_decontam_asm.fa ./red_in/

Red -gnm red_in/ -rpt red_out/ -msk red_out/
cp red_out/aedes_final_decontam_asm.msk
./aedes_final_decontam_mask.fa
```

Prediction of rRNA genes

```
# (Barrnap 0.9)

barrnap --kingdom euk --threads 6 --lencutoff 0.95 --reject 0.9 --
evalue 0.0000000000000001 --outseq barrnap.fa
aedes_final_decontam_mask.fa > barrnap.gff3 2> barrnap.log
```

Prediction of tRNA genes

```
# (ARAGORN 1.2.38) and convert the results into a GFF file

aragorn -m -t -l -gc1 -w -seq -fasta -o
final_decontam_mask_aragorn.txt aedes_final_decontam_mask.fa

awk -F "|" '/pilon/ {close(F); ID=$1; gsub("^>", "", ID);
F=ID".txt"} {print >> F}' final_decontam_mask_aragorn.txt
grep "0 genes found" *pilon*txt | cut -f 1 -d ":" | xargs rm
for i in $(ls *pilon*txt)
do
grep tRNA $i | grep -v ">" | sed -e "s/]//" -e "s/,/ /" | while
read line
do
id=$(echo $line | cut -f 2,6 -d " " | sed 's/ //')
if [[ $line == *c[* ]]; then
start=$(echo $line | cut -f 4 -d " ")
end=$(echo $line | cut -f 3 -d " " | sed 's/c\['//)
else
start=$(echo $line | cut -f 3 -d " " | sed 's/\['//)
end=$(echo $line | cut -f 4 -d " ")
fi
if [[ "$start" -ge "$end" ]]; then
strand="-"
else
strand="+"
fi
echo "$i" "aragorn" tRNA $start $end "." $strand "." "Name=${id}"
| sed -e "s/ /\t/g" -e "s/.txt//"
done
done | sed '1i ##gff-version 3' > aragorn.gff
```

```
rm *pilon*txt
```

Gene prediction

```
# Gene prediction using ab initio and homology-based methods
(BRAKER 3.0.2)

# Download the orthoDB of arthropods

wget -c https://bioinf.uni-greifswald.de/bioinf/partitioned_odb11/
Arthropoda.fa.gz
gzip -d Arthropoda.fa.gz

# Run gene prediction

singularity exec braker3.sif braker.pl --
genome=aedes_final_decontam_mask.fa --prot_seq=Arthropoda.fa --
species=aedkor --useexisting --threads 30 --
workingdir=aedes_braker3_odb11 --verbosity=3 --AUGUSTUS_ab_initio

# Merge the ab initio and homology-based gene sets

# Rename sequences to avoid duplicated gene names in the
functional annotation

awk '{if($1~">"){gsub("g","abi");print $1} else {print $0}}'
Augustus/augustus.ab_initio.aa > abinitio_newnames.aa
awk '{if($1~">"){gsub("g","hom");print $1} else {print $0}}'
braker.aa > hombased_newnames.aa

# Cluster those genes that have 100% identical sequences from the
two different prediction methods (CD-HIT 4.7)

cat abinitio_newnames.aa hombased_newnames.aa > merged_newnames.aa
cd-hit -i merged_newnames.aa -c 1 -G 0 -aL 1.0 -aS 1.0 -o
cdhit_merged_newnames.aa

# Repeat the same step with the old names to have the sequence
names in the exact same order

cat Augustus/augustus.ab_initio.aa braker.aa > merged_oldnames.aa
cd-hit -i merged_oldnames.aa -c 1 -G 0 -aL 1.0 -aS 1.0 -o
cdhit_merged_oldnames.aa

# Create a table with the sequence name pairs

grep '^>' cdhit_merged_newnames.aa | sed 's/>//g' >
cdhit_merged_newnames.list
grep '^>' cdhit_merged_oldnames.aa | sed 's/>//g' >
cdhit_merged_oldnames.list
paste cdhit_merged_oldnames.list cdhit_merged_newnames.list >
code_for_genenames.tab
```

Functional annotation

```
# (PANNZER2) - The webserver can be reached
[here](http://ekhidna2.biocenter.helsinki.fi/sanspanz/)

# Get the sequence names from the genes (transcripts) having
functional description or gene onthology as a result of functional
annotation

cat DE.out GO.out | cut -f 1 | sort | uniq | grep abi >
func_abi_transcripts.list
cat DE.out GO.out | cut -f 1 | sort | uniq | grep hom >
func_hom_transcripts.list

# Get the original names of the sequences

grep -w -f func_abi_transcripts.list ../code_for_genenames.tab |
cut -f 1 | sort > func_abi_transcripts_oldnames.list
grep -w -f func_hom_transcripts.list ../code_for_genenames.tab |
cut -f 1 | sort > func_hom_transcripts_oldnames.list

# Extract the structural annotation information for the
functionally annotated sequences

grep -w -f func_abi_transcripts_oldnames.list
../Augustus/augustus.ab_initio.gtf > func_abi.gtf
sed 's/.t[0-9]*//g' func_abi_transcripts_oldnames.list | sort |
uniq > func_abi_genes_oldnames.list
grep -w -f func_abi_genes_oldnames.list
../Augustus/augustus.ab_initio.gtf | awk '$3 == "gene"' >>
func_abi.gtf

grep -w -f func_hom_transcripts_oldnames.list ../braker.gtf >
func_hom.gtf
sed 's/.t[0-9]*//g' func_hom_transcripts_oldnames.list | sort |
uniq > func_hom_genes_oldnames.list
grep -w -f func_hom_genes_oldnames.list ../braker.gtf | awk '$3 ==
"gene"' >> func_hom.gtf
## Rename sequences (there are scaffold names starting with "ctg"
which are renamed so we have to correct those)
sed 's/g\([0-9]\)/abi\1/g' func_abi.gtf | sed 's/ctabi/ctg/g' >
func_abi_newnames.gtf
sed 's/g\([0-9]\)/hom\1/g' func_hom.gtf | sed 's/cthom/ctg/g' >
func_hom_newnames.gtf

# Merge the annotation files from the two different gene
prediction methods as well as the other sequence features
predicted in the genome

cat func_abi_newnames.gtf func_hom_newnames.gtf >
pannzer_all_de_go.gtf

# Make gff3 from gtf (GenomeTools 1.5.10)
```

```
gt gtf_to_gff3 -tidy yes -o pannzer_all_de_go.gff3
pannzer_all_de_go.gtf &> stdout
```

```
# Sort and tidy gff3
```

```
gt gff3 -sort yes -retainids yes -tidy yes -addintrons yes -o
pannzer_all_de_go_sorted.gff3 pannzer_all_de_go.gff3
```

```
# Extract the functionally annotated protein and gene sequences
(SeqKit 2.3.0)
```

```
cd ../
cat pannzer/func_abi_transcripts.list
pannzer/func_hom_transcripts.list | seqkit grep -f -
cdhit_merged_newnames.aa > functionally_annotated_proteins.fasta

seqkit grep -f pannzer/func_abi_transcripts_oldnames.list
Augustus/augustus.ab_initio.codingseq >
func_abi_transcripts_oldnames.cds
awk '{if($1~">"){gsub("g","abi");print $1} else {print $0}}'
func_abi_transcripts_oldnames.cds > func_abi_transcripts.cds
seqkit grep -f pannzer/func_hom_transcripts_oldnames.list
braker.codingseq > func_hom_transcripts_oldnames.cds
awk '{if($1~">"){gsub("g","hom");print $1} else {print $0}}'
func_hom_transcripts_oldnames.cds > func_hom_transcripts.cds
cat func_abi_transcripts.cds func_hom_transcripts.cds >
functionally_annotated_cds.fasta
```

```
# Create full annotation table
```

```
gt gff3 -sort yes -retainids yes -tidy yes -o barrnap_sorted.gff3
barrnap.gff3
gt gff3 -sort yes -retainids yes -tidy yes -o aragorn_sorted.gff3
aragorn.gff
gt merge -retainids yes -tidy yes -o
pannzer_barrnap_aragorn_merged.gff3
pannzer/pannzer_all_de_go_sorted.gff3 barrnap_sorted.gff3
aragorn_sorted.gff3
```

```
# Annotation transfer and screen resistance genes
```

```
liftoff -g
GCF_002204515.2/ncbi_dataset/data/GCF_002204515.2/genomic.gff -o
aedes_final_decontam_mask_litfoff.gff aedes_final_decontam_mask.fa
GCF_002204515.2/ncbi_dataset/data/GCF_002204515.2/GCF_002204515.2_
AaegL5.0_genomic.fna
```

Ortholog finding and phylogenetic analysis

```
# Download all proteomes available in the Culicidae family (16 Jun
2023; datasets 12.32.0)

datasets download genome taxon culicidae --include protein --
filename culicidae.zip && culicidae.zip
cp functionally_annotated_proteins.fasta
culicidae/Aedes_koreicus.faa

# Rename the proteomes according to their species name

cd culicidae/
for i in `ls -d GC*`
do
paste <(echo ${i}) <(head -n1 ${i}/*.fna | cut -d '[' -f 2 | cut -
d ']' -f 1 | sed 's/ /_/g') >> code_for_names.tab
done

while read dirname newname
do
mv ${dirname}/protein.faa ./${newname}.faa
rm -r $i
done < code_for_names.tab

# Find orhtologs and reconstruct phylogenetic tree (OrthoFinder
2.5.5)

orthofinder.py -t 12 -a 12 -f culicidae/
```
