# Supplementary material for: The updated genome of the Hungarian population of Aedes koreicus
Source: Sci Rep. 2024 Mar 30;14:7545. doi: 10.1038/s41598-024-58096-6 (PMC10981705; doi:10.1038/s41598-024-58096-6)
Supplement: Supplementary file 2 — Supplementary Information 2. [file 41598_2024_58096_MOESM2_ESM.pdf]

Supplementary Table 1. Contiguity statistics of polished assemblies obtained with QUAST after the different steps outlined in the Methods.

Assembly	aedes_final_asm_polished	aedes_final_decontam_asm	aedes_nuc_masurca_polished	aedes_nuc_masurca_pseudohap_polished	aedes_nuc_merged_pseudohap_polished	aedes_nuc_nd_polished	aedes_nuc_nd_pseudohap_polished
# contigs (>= 0 bp)	6,344	6,099	10,588	10,509	6,344	9,809	9,798
# contigs (>= 1000 bp)	6,344	6,099	10,588	10,509	6,344	9,809	9,798
# contigs (>= 5000 bp)	6,344	6,099	10,569	10,488	6,344	9,809	9,798
# contigs (>= 10000 bp)	6,338	6,094	10,438	10,359	6,338	9,802	9,790
# contigs (>= 25000 bp)	6,014	5,853	9,327	9,275	6,055	9,342	9,283
# contigs (>= 50000 bp)	4,570	4,525	7,087	7,042	4,610	5,663	5,540
Total length (>= 0 bp)	1,102,764,628	1,100,025,007	1,186,008,384	1,175,109,693	1,117,038,154	861,327,324	848,911,962
Total length (>= 1000 bp)	1,102,764,628	1,100,025,007	1,186,008,384	1,175,109,693	1,117,038,154	861,327,324	848,911,962
Total length (>= 5000 bp)	1,102,764,628	1,100,025,007	1,185,932,938	1,175,026,496	1,117,038,154	861,327,324	848,911,962
Total length (>= 10000 bp)	1,102,722,465	1,099,986,680	1,184,913,255	1,174,027,388	1,116,993,727	861,265,707	848,844,669
Total length (>= 25000 bp)	1,095,951,231	1,094,856,870	1,164,604,509	1,154,363,766	1,110,998,025	851,334,543	837,953,812
Total length (>= 50000 bp)	1,042,902,999	1,045,717,258	1,080,963,971	1,070,992,523	1,057,728,786	712,950,331	697,667,952
# contigs	6,344	6,099	10,585	10,506	6,344	9,809	9,798
Largest contig	3,240,226	3,269,480	1,689,014	1,683,355	3,269,480	2,205,367	2,172,861

Assembly	aedes_final_asm_polished	aedes_final_decontam_asm	aedes_nuc_masurca_polished	aedes_nuc_masurca_pseudohap_polished	aedes_nuc_merged_pseudohap_polished	aedes_nuc_nd_polished	aedes_nuc_nd_pseudohap_polished
Total length	1,102,764,628	1,100,025,007	1,186,000,800	1,175,102,307	1,117,038,154	861,327,324	848,911,962
GC (%)	39.65	39.67	39.67	39.68	39.65	39.60	39.62
N50	321,124	329,610	167,606	166,603	324,449	108,532	107,467
N90	71,932	74,757	53,155	53,119	72,645	40,369	39,705
auN	463,040	472,147	258,370	257,539	467,798	243,228	240,551
L50	916	896	1,885	1,874	919	1,722	1,712
L90	3,739	3,626	6,824	6,783	3,752	7,044	7,023
# N's per 100 kbp	2	3	10	7	3	0	0

Supplementary Table 2. Potential resistance genes as identified after using annotation transfer. The reference is the publicly available genome of *Aedes albopictus* and the target is the updated draft genome *Aedes koreicus*.

Gene ID	NCBI Gene description	NCBI gene ID	Reference scaffold	Start position in reference	End position in reference	Target scaffold	Start position in target	End position in target	Functional annotation of overlapping transcripts predicted using BRAKER3 as output by PANNZER
aael012918	puromycin-sensitive aminopeptidase	5576957	NC_035108.1	160,240,665	160,286,123	jcf7180000112576	90,220	112,809	Aminopeptidase
*ace*1	acetylcholinesterase	5578456	NC_035109.1	161,486,025	161,871,375	ctg039070	257,954	279,949	Acetylcholinesterase, Carboxylic ester hydrolase

Gene ID	NCBI Gene description	NCBI gene ID	Reference scaffold	Start position in reference	End position in reference	Target scaffold	Start position in target	End position in target	Functional annotation of overlapping transcripts predicted using BRAKER3 as output by PANNZER
*ABCA*3	ATP-binding cassette sub-family A member 3	5576676	NC_035108.1	94,093,797	94,103,717	ctg063300	336,413	341,870	ATP-binding cassette sub-family A member 3
*cceae*3a	esterase B1	5566024	NC_035108.1	174,400,171	174,416,020	-	-	-	-
cuticle protein	cuticle protein	5572415	NC_035107.1	276,097,787	276,098,756	jcf7180000110969	467,620	468,224	Cuticular protein 73
cuticle protein	cuticle protein	5572415	NC_035108.1	67,627,826	67,628,518	jcf7180000111681	160,891	161,347	Pupal cuticle protein 78e
cuticle protein	cuticle protein	5572415	NC_035108.1	288,501,640	288,502,453	-	-	-	-
cuticle protein 7	cuticle protein 7	5565393	NC_035108.1	288,501,640	288,502,453	-	-	-	-
cuticle protein CP14.6	cuticle protein CP14.6	5570386	NC_035108.1	67,627,826	67,628,518	jcf7180000111681	160,891	161,347	Cuticle protein CP14.6
*cyp*6bb2	probable cytochrome P450 6a14	5565578	NC_035107.1	271,328,495	271,330,383	ctg028580	14,728	16,087	Putative cytochrome
*cyp*6cb1	probable cytochrome P450 6a14	5571382	NC_035107.1	58,798,630	58,800,576	-	-	-	-
*cyp*9j10	probable cytochrome P450 9f2	5564750	NC_035109.1	368,502,058	368,504,167	-	-	-	-

Gene ID	NCBI Gene description	NCBI gene ID	Reference scaffold	Start position in reference	End position in reference	Target scaffold	Start position in target	End position in target	Functional annotation of overlapping transcripts predicted using BRAKER3 as output by PANNZER
*cyp*9j19	probable cytochrome P450 9f2	5579933	NC_035109.1	368,517,401	368,519,262	-	-	-	-
*cyp*9j24	probable cytochrome P450 9f2	5564763	NC_035109.1	368,585,862	368,587,982	jcf7180000109840	800,637	802,198	-
*cyp*9j26	probable cytochrome P450 9f2	5564749	NC_035109.1	368,609,784	368,611,605	jcf7180000109840	788,091	789,769	Cytochrome
*cyp*9j28	cytochrome P450 9e2	5564751	NC_035109.1	368,627,096	368,628,939	jcf7180000109840	802,853	804,461	Putative cytochrome
*cyp*9j32	probable cytochrome P450 9f2	5571141	NC_035108.1	240,837,377	240,839,772	jcf7180000109840	747,506	748,638	Cytochrome
*cyp*9m6	cytochrome P450 9b2	5569822	NC_035108.1	447,504,779	447,506,604	-	-	-	-
*gstd*4	glutathione S-transferase 1	5568346	NC_035107.1	301,212,310	301,213,169	-	-	-	-
*gstd*6	glutathione S-transferase 1	5573540	NC_035107.1	301,236,089	301,237,254	-	-	-	-
ketohexokinase	ketohexokinase	5567861	NC_035109.1	2,698,467	2,718,620	ctg076250	6,071	7,274	Phospholipid/ glycerol acyltransferase domain-containing protein

Gene ID	NCBI Gene description	NCBI gene ID	Reference scaffold	Start position in reference	End position in reference	Target scaffold	Start position in target	End position in target	Functional annotation of overlapping transcripts predicted using BRAKER3 as output by PANNZER
modifier of *mdg*4	modifier of mdg4	5573536	NC_035107.1	301,364,360	301,784,078	jcf7180000116134	386,922	405,025	Modifier of mdg4 isoform X2
multidrug resistance-associated protein 1	multidrug resistance-associated protein 1	5565387	NC_035108.1	289,194,273	289,251,024	-	-	-	-
muscle calcium channel subunit alpha-1	muscle calcium channel subunit alpha-1	5574639	NC_035108.1	225,875,648	225,962,971	jcf7180000109321	531,277	532,217	Muscle calcium channel subunit alpha-1
nav	sodium channel protein para	5567355	NC_035109.1	315,926,360	316,405,639	jcf7180000109739	142,336	358,654	Ion transport domain-containing protein, Voltage-dependent para-like sodium channel, Voltage-gated sodium channel

Gene ID	NCBI Gene description	NCBI gene ID	Reference scaffold	Start position in reference	End position in reference	Target scaffold	Start position in target	End position in target	Functional annotation of overlapping transcripts predicted using BRAKER3 as output by PANNZER
potassium voltage-gated channel protein Shaker	potassium voltage-gated channel protein Shaker	5572028	NC_035107.1	5,749,599	6,294,412	jcf7180000121653	83,373	166,621	Potassium voltage-gated channel protein Shaker
rdl	gamma-aminobutyric acid receptor subunit beta	5570466	NC_035108.1	41,628,484	41,861,946	jcf7180000160213	106,074	170,584	GABA-gated chloride channel, Gaba receptor invertebrate, Gamma-aminobutyric acid receptor subunit beta, Gamma-aminobutyric acid receptor subunit beta, Neurotransmitter-gated ion-channel transmembrane

Gene ID	NCBI Gene description	NCBI gene ID	Reference scaffold	Start position in reference	End position in reference	Target scaffold	Start position in target	End position in target	Functional annotation of overlapping transcripts predicted using BRAKER3 as output by PANNZER
									domain-containing protein
sodium leak channel non-selective protein	sodium leak channel non-selective protein	5579947	NC_035109.1	169,273,369	169,346,164	ctg017590	139,864	159,462	Sodium leak channel non-selective protein
uncharacterized LOC5575776	uncharacterized LOC5575776	5575776	NC_035109.1	125,588,140	125,711,451	jcf7180000115313	102,587	206,968	chitin synthase
